# Sexual dimorphism in *Homo erectus* inferred from 1.5 Ma footprints near Ileret, Kenya

**DOI:** 10.1038/s41598-019-44060-2

**Published:** 2019-05-22

**Authors:** Brian Villmoare, Kevin G. Hatala, William Jungers

**Affiliations:** 1 Department of Anthropology, University of Nevada Las Vegas, 89154-5003 Las Vegas, NV USA; 2 Department of Biology, Chatham University, 15232 Pittsburgh, PA USA; 3 Department of Anatomical Sciences, Stony Brook University, Stony Brook, 11794-8081 New York, USA; 4 Association Vahatra, BP 3972, 101 Antananarivo, Madagascar

**Keywords:** Sexual selection, Palaeontology

## Abstract

Sexual dimorphism can be one of the most important indicators of social behavior in fossil species, but the effects of time averaging, geographic variation, and differential preservation can complicate attempts to determine this measure from preserved skeletal anatomy. Here we present an alternative, using footprints from near Ileret, Kenya, to assess the sexual dimorphism of presumptive African *Homo erectus* at 1.5 Ma. Footprint sites have several unique advantages not typically available to fossils: a single surface can sample a population over a very brief time (in this case likely not more than a single day), and the data are geographically constrained. Further, in many cases, the samples can be much larger than those from skeletal fossil assemblages. Our results indicate that East African *Homo erectus* was more dimorphic than modern *Homo sapiens*, although less so than highly dimorphic apes, suggesting that the Ileret footprints offer a unique window into an important transitional period in hominin social behavior.

## Introduction

One of the most important proxies for social behavior in a fossil species is its sexual dimorphism, which has the potential to provide a unique window into the social structure of an extinct population^1–5^. Considerable research has been invested in determining what information can be inferred from different patterns of sexual dimorphism^6–15^, as well as ensuring that statistical inferences of dimorphism in fragmentary fossil samples are accurate^16–20^.

Many hominin species are not sufficiently sampled to make inferences about patterns of sexual dimorphism in their fossil populations, but certain well-represented species such as *Australopithecus afarensis*, *A*. *africanus*, *Paranthropus boisei*, *P*. *robustus*, *Homo erectus*, *H*. *heidelbergensis*, and *H*. *neanderthalensis* have all been studied in the context of their sexual dimorphism^4,16–31^. Most analyses have determined that Pliocene and some early Pleistocene hominins (*A*. *afarensis*, *A*. *africanus*, *P*. *boisei*, *P*. *robustus*) appear to have been highly dimorphic, with patterns of dimorphism similar to that seen in extant gorillas^4,17,25,27–30^. These results have implied gorilla-like social structures, with polygyny and high levels of male-male agonistic competition, in these Pliocene and early Pleistocene hominin taxa (but see^18,19^ for a contrary position, and^31^ for a more nuanced view).

Prior to the discovery of KNM-ER 42700^32^, and recent work on postcranial fossils^33^ (and see below), most analyses of variation in *Homo erectus* had identified only a relatively modest degree of dimorphism, similar to the pattern observed in modern humans^3,4,34,35^. This low degree of variation stood in sharp contrast to the higher degree of dimorphism seen in *Australopithecus* and *Paranthropus* (see above). This apparent transition from a primitive ape-like condition to a more modern human-like pattern was among a suite of traits allying this species with later *Homo* under many adaptive scenarios (e.g., Out of Africa I^3,36^). With the discovery of KNM-ER 42700, a small calvarium overlapping in size with earlier species of *Homo*, the range of size variation in the species was extended considerably, to such a degree that the discoverers proposed the retention of a primitive, high degree of size dimorphism for *H*. *erectus*^32^.

As a species, *H*. *erectus* shows considerable temporal and geographic variation and, as Plavcan^5^ notes, only the African sample (in which he includes Dmanisi) shows a sufficiently high degree of variation to falsify the hypothesis that the species had acquired the relatively monomorphic human-like condition. In his analysis, the high variation in the African sample was driven by the small size of KNM-ER 42700 and the large size of OH 9 (which are separated geographically by 800 kilometers, and temporally by more than 300,000 years), although the Dmanisi sample also increases the variation within *H*. *erectus* considerably^37^. The Chinese and Javan samples show a level of variation in cranial measures very similar to that seen in modern humans^37,38^. The different patterns in the African and Asian samples raise the possibility of a change in patterns of dimorphism, and potentially social behavior, in the *H*. *erectus* lineage over time and/or across different geographic regions. A recent analysis by Grabowski *et al*.^33^ has made the picture of sexual dimorphism in *H*. *erectus* even fuzzier, as they found a level of variation in postcranial measures, across both African and Asian samples that suggested a level of body size dimorphism that exceeded that observed in modern humans.

However, skeletal fossils are not the only source of evidence for size dimorphism and, in turn, inferred early hominin social behavior. Assemblages of footprints are now known for an increasing number of fossil hominin taxa and they represent an arguably more direct path for accessing past hominin (and other animal) social behaviors. Footprint surfaces can offer exceptional levels of temporal and spatial constraint, representing snapshots of hominins who passed through the same location within hours to days of each other and probably lived in immediate proximity to each other, if not within the same social group. Meanwhile, a skeletal fossil sample might come from multiple countries/continents, and from geological contexts that span tens or hundreds of thousands of years. Due to the remarkable constraints on error from space- and time-averaging, the context of footprint assemblages offers a potentially more powerful and more direct approach for testing hypotheses regarding size dimorphism within specific hominin populations.

In terms of estimating body size dimorphism, these trace fossils offer some advantages, and some disadvantages, relative to skeletal fossils. The main disadvantage is that the data represent a relatively limited anatomical region (the foot) from which to make inferences. However, considering the typically fragmentary nature of skeletal fossils, perhaps this difference in anatomical coverage is not substantial. Additionally, footprints can only form in specific environments and they are relatively fragile, potentially leading to a different set of taphonomic biases. Very unlike skeletal fossils, the potential also exists for repeatedly sampling the same individuals multiple times within a footprint assemblage. Finally, while rates of fusion of skeletal elements offer methods for age determination that are independent of body size, allowing one to rather confidently exclude juvenile specimens when estimating size dimorphism, there is no analogous method for distinguishing small adult footprints from those of larger juveniles.

However, footprints also offer several advantages compared with skeletal fossils. One of the most obvious advantages is sample size; hominin footprints are not common, but when footprint surfaces are found they can often sample multiple individuals. Perhaps most importantly for evaluating social behavior, depositions of footprints occur over very short periods of time in geographically constrained areas, such that one can directly view multiple individuals who lived in close proximity to each other, and perhaps regularly interacted during their lifetimes. Few fossil sites offer large samples in geographically small and temporally constrained locales (the Atapuerca^39^, or Rising Star (Dinaledi chamber)^40^ fossil sites are the perhaps most notable exceptions). Even the most constrained fossil assemblages are still subject to a level of time-averaging that usually exceeds that of a footprint surface.

Analyses of hominin footprints have tended to focus on biomechanical and anatomical inferences (e.g.^41–54^ although see^55–57^). In this manuscript our focus is instead on using these data to estimate size dimorphism and ultimately test hypotheses related to hominin social behavior. Here we examine several early hominin footprint localities and attempt to infer degrees of sexual dimorphism in the populations that made those footprints. We keep a specific eye towards evaluating sexual dimorphism inferred from sets of 1.5 Ma footprints discovered near Ileret, Kenya, which are hypothesized to represent *H*. *erectus*^55–57^.

## Materials

Data were compiled from published fossil hominin footprint sites from different times and places, including Laetoli, Tanzania (~3.66 Ma^58^), Ileret, Kenya (~1.5 Ma^48^), Willandra Lakes, Australia (~23–19 ka^59^), Engare Sero, Tanzania (~19.1–5.8 ka^60^), and Walvis Bay, Namibia (~500–400 BP^61^). Data were derived from our own work on the Ileret, Kenya and Engare Sero footprint assemblages^52,56,57,60^ and published measurements from the other localities (Laetoli^53,62,63^, Willandra Lakes^59^, Walvis Bay^61^). Because of the potential for repeatedly sampling the same individual, the fossil data sets were each reduced by calculating a mean footprint length for each identifiable trackway (i.e., sequences of consecutive steps taken by the same individual). Isolated footprints that were not clearly associated with any trackways were also included in our analysis, and treated as if they represented unique individuals. This procedure does not eliminate the possibility of repeatedly sampling an individual, but it reduces the influence of repetition to the extent that is currently possible. Because we are interested in sexual dimorphism, we used only adult tracks from the three relatively recent (<25,000 years) modern human footprint sites (Willandra Lakes, Engare Sero, Walvis Bay), excluding any tracks that were likely or possibly produced by children. For the Willandra Lakes assemblage, children’s footprints were identified by previous authors based on size variation patterns, and/or body size interpolations, and footprints less than 22 cm in maximum length were considered likely to have been produced by juveniles^59^. Individual age estimates have not yet been published for the Engare Sero or Walvis Bay assemblages analyzed here, so we instead applied the same criteria as Webb^59^, excluding from our analysis any tracks that were less than 22 cm long. Supplemental analyses that include all tracks from each of these sites are presented in the Appendix.

For the cases of the Ileret footprints, two trackways had average lengths that were slightly below this 22 cm threshold (20.5 and 21 cm). However, given that published estimates of stature derived from long bones attributed to *H*. *erectus* are, on average, lower than the average statures observed in either of our modern human comparative samples (153.7 cm^3^ compared with 169.5 cm [US Army] and 169.1 cm [Daasanach]), we did not feel that a rigid cutoff to exclude an extra 1.5 cm of potential foot/footprint length variation was justified in this Pleistocene sample. Regarding the Laetoli footprints, Tuttle *et al*. demonstrated that the lengths of the smallest set of tracks (from the G1 trackway) still fall within the range of foot lengths observed in short-statured adults from a modern habitually unshod population, whose foot indices represent the closest modern match for the Laetoli G1 tracks^64^. For this reason, we felt it was also prudent to include the G1 trackmaker in our estimates of adult size dimorphism derived from the Laetoli footprints. However, supplemental analyses presented in the Appendix describe the results while excluding Laetoli trackway G1, and while excluding the smaller Ileret prints.

Comparative data were taken from two samples of roughly equally represented mixed-sex modern humans, and several samples of directly measured great ape foot lengths. In 2011 and 2012, data from 366 footprints created by 29 Daasanach adults (14 females, 15 males) were collected, along with measurements of subjects’ heights, weights, stride lengths, and foot sizes^54^. As with the fossil assemblages, an average footprint length for each subject was computed within this data set. The second human sample was compiled from a US Army study of foot size^65^. A random sample of foot length measurements from 2000 individual adults (1000 female, 1000 male) were extracted from this sample for use in our analyses. Samples of gorilla (*Gorilla gorilla gorilla*; n = 31 individuals) and chimpanzee (*Pan troglodytes troglodytes*; n = 37) foot lengths were collected from field measurement records of wild-shot individuals housed at the Powell-Cotton Museum. A smaller comparative sample (n = 8) of bonobo (*Pan paniscus*) foot length data from the literature^66^ was also included.

## Methods

One difficulty with examining sexual dimorphism in the fossil record is that it is extremely difficult to be completely confident of sex attributions of fossils, even in apparently dimorphic species. Unlike with extant taxa, a direct index of male to female average size cannot be calculated, since we cannot be sure of correctly attributing every fossil. Even in highly dimorphic species, there can be some degree of overlap between males and females in many anatomical measures^25,27,67^. Since fossils cannot be directly attributed to sex, multiple statistics derived from a pooled sample have been used to infer some measure of sexual dimorphism, including the coefficient of variation (CV; calculated as 100 × standard deviation/mean), min/max, R% (max-min/mean), and mean method ratio^17,29,68–71^. In general, most analyses have concluded that the mean method ratio (assignment of specimens to sex based on position above or below the sample mean) provides the most reliable estimator of size dimorphism^29,68,70^, although all of these statistics can overestimate the true levels of dimorphism in species with low dimorphism^68^. We focus on the mean method ratio as the primary test statistic for comparing size dimorphism between modern and fossil data sets. However, given its frequent use as a measure of size variation among fossil taxa, we also present results using the CV as a test statistic. We compute and report min/max and R% values for each of our samples, although we do not use these as test statistics given their far less frequent use.

We use measures of fossil footprint length as proxies for foot size. The Daasanach footprint length, US Army foot length, and gorilla foot length samples were used as reference samples against which the fossil footprint samples were evaluated. We report comparative data from samples of *Pan troglodytes* and *Pan paniscus* but we do not use these samples for direct hypothesis testing because they involve some potentially confounding issues. The chimpanzee data was sexually biased (n_female_ = 31, n_male_ = 8), and the bonobo dataset was relatively small (n = 8). So, while these data provide potentially useful comparisons of variation, they are less applicable for hypothesis testing.

The extant comparative data also allowed us to examine the relationship between foot size and body size, to understand how our dimorphism estimates derived from footprints might relate to estimates derived from other parts of the body. Although body mass data were not available for the ape sample, stature estimations were made from their complete skeletons (heel – vertex measurements). Statures were measured directly for the 29 adults within the Daasanach sample. From the US Army data set, we randomly sampled 100 20-year old soldiers, evenly balanced among males and females, from whom both foot lengths and statures were measured. We plotted foot lengths and statures for these samples (footprint lengths in the case of the Daasanach sample) and we used regression analyses to examine the statistical relationships between these variables within each group.

Hypothesis testing for sexual dimorphism involved comparing the dimorphism estimates for each of the fossil samples to bootstrapped comparative samples of humans and gorillas. The human and gorilla samples represent opposite ends of the spectrum of sexual dimorphism observed in extant large-bodied hominoids. In each iteration of the bootstrapping protocol, samples equal in size to each fossil sample were randomly drawn from the comparative data, and the dimorphism measures were calculated. This was repeated 1000 times, a distribution of comparative measures was generated, and the fossil measures were compared to this distribution. This allowed us to calculate the probability of drawing the observed dimorphism measures in a given fossil sample from any of the comparative samples. As we were testing whether the fossil footprint samples showed evidence of greater sexual dimorphism than is seen in the feet of the modern humans, or lesser than observed in the feet of gorillas, the tests were one-tailed, although in different directions in these two cases.

Because the Ileret hominin footprint sample includes data from multiple track surfaces that are non-continuous and stratigraphically distinct, analyses of that assemblage were split into two parts. In one, we computed and compared dimorphism metrics using only data from a single track surface, the FwJj14E Upper Footprint Layer (FwJj14E UFL). This surface spans 54 m^2^, and includes a total of 64 hominin footprints^57^. Eight distinct trackways and eight isolated footprints that could not be reasonably connected to any trackways were included in this analysis as 16 distinct ‘individuals’. Sedimentological studies and modern taphonomic experiments have suggested that these hominin footprints on the FwJj14E UFL were made in a span of 1.3 days or less^55^, supporting the hypothesis that they sample individuals who lived within the same population. Therefore, analyses of this site may provide the most preferable scenario for estimating within-population dimorphism. These data were compared directly with the other fossil hominin footprint sites mentioned above, as each of those sites samples only a single track surface.

However, we also recognized the possibility that the FwJj14E sample could represent a type of group behavior with inherent biases that could affect size dimorphism estimates. Certain behaviors may be divided between sexes (e.g., foraging behaviors are often sexually divided in modern humans^70^) and dimorphism estimates could therefore be skewed if a set of footprints captures predominantly a single sex. For this reason, we re-ran all analyses while also including data from four additional track surfaces known near Ileret (ET-2013-1A-FE1, ET-2013-1A-FE3, ET-2014-3-FE8, FwJj14E Lower Footprint Layer^57^). This expanded sample is still relatively time-constrained, as the five sites all lie in stratigraphic sequence between volcanic tuffs that differ in age by about 20 ka^57^, but there is no way of knowing whether these footprints may sample the same continuous population (and given their different stratigraphic positions, they probably do not). Our reason for expanding the sample in this manner was that it might uncover sex bias in the FwJj14E UFL assemblage, if dimorphism estimates are elevated by including data from other Ileret surfaces. Also, if the FwJj14E UFL is sex-biased then a broader analysis of tracks from multiple surfaces may provide a more accurate dimorphism estimate for early Pleistocene East African *H*. *erectus*, if on the grounds of parsimony we assume that all of the Ileret tracks were made by *H*. *erectus* (see^56^).

## Results

**Figure 1:**
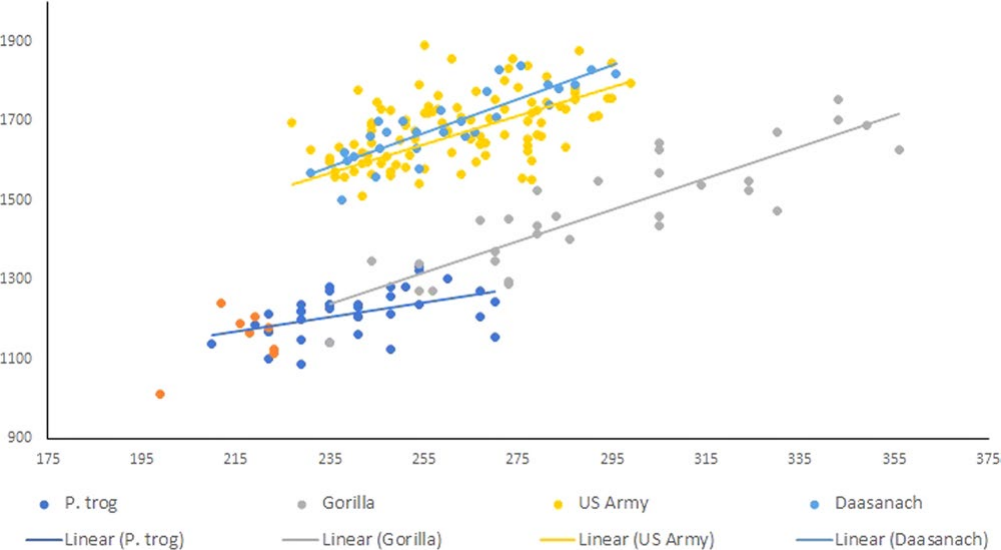
Scatterplot of foot lengths and statures for bonobos (red), chimpanzees (dark blue), gorillas (gray), and modern humans (yellow – US Army sample, light blue – Daasanach sample). Linear regression fits are indicated by the solid lines corresponding to each group. Within chimpanzees, gorillas, and both modern human groups, there are statistically significant relationships between foot size and stature, suggesting that foot or footprint size dimorphism should closely track overall body size dimorphism for each of these samples. Due to the small size of the *Pan paniscus* sample, regression analyses were not performed for this group (see Table 2).

In each of the modern taxonomic groups (*Pan*, *Gorilla*, *H*. *sapiens*), there was a strong statistical relationship between foot length and stature. Results of the regression analyses for these individual samples are presented in Table 1 (see also Fig. 1). These results provide reasonable confidence that inferences made from foot size dimorphism will closely resemble similar inferences from overall body size dimorphism. Sexual dimorphism indices, calculated using the mean method quotient (mean male size/mean female size), are reported in Table 2.

**Table 1:** Statistics for regressions of stature as predicted by foot size for all modern samples. All regressions are statistically significant.

	Slope	95% CI+/−	*p* value	r^2^	*p* value
*Pan troglodytes*	1.798	0.596	0.0048	0.211	<0.0005
*Gorilla*	3.933	0.419	<0.00001	0.752	<0.0001
Humans - Army	4.067	0.264	<0.00001	0.708	<0.0001
Humans - Daasanach	4.317	0.497	<0.00001	0.736	<0.0001
*Pan* pooled	2.140	0.501	0.00011	0.302	0.0001
Humans pooled	4.109	0.234	<0.00001	0.709	<0.0001

The small sample size of the *Pan paniscus* precluded regression, so for one analysis all *Pan* was pooled.

**Table 2:** Indices of sexual dimorphism, calculated using the mean method quotient (mean male size/mean female size), for stature and foot length within each of the extant comparative samples.

	Stature dimorphism	Foot length dimorphism
*Pan troglodytes*	1.044	1.015
*Pan paniscus*	1.009	1.065
*Gorilla*	1.192	1.148
Modern *H*. *sapiens*	1.103	1.066

**Figure 2:**
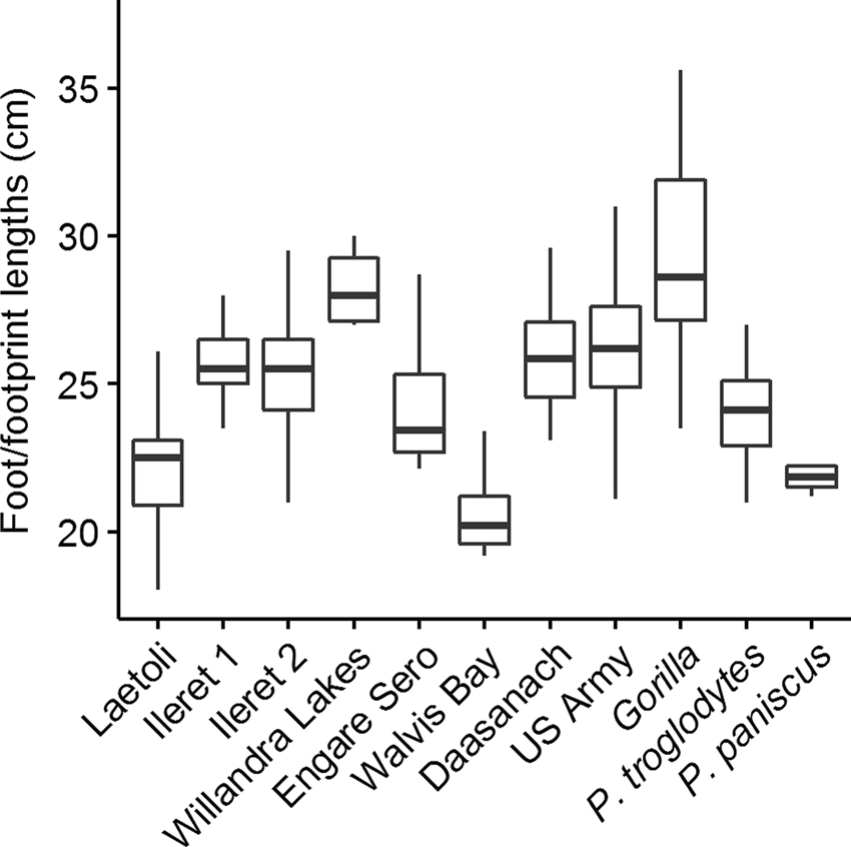
Boxplots of footprint and foot lengths for the fossil and extant samples used in this study using raw data. Note the relatively high level of variation in the *Gorilla* sample. Ileret 1 corresponds to the Ileret FwJj14E UFL sample, while Ileret 2 corresponds to the pooled Ileret sample.

**Figure 3:**
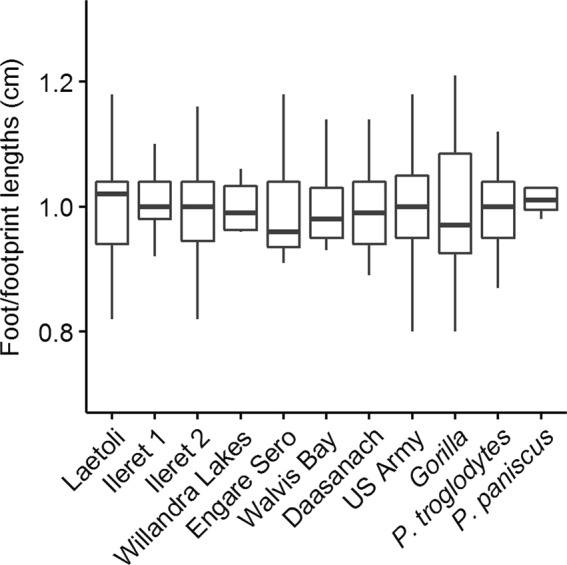
Boxplots of footprint and foot lengths in mean-adjusted format for the fossil and extant samples used in this study. Mean-adjusted sizes are calculated by dividing individual footprint/foot lengths by the within-sample mean. Note the relatively high level of variation in the *Gorilla* sample. Ileret 1 corresponds to the Ileret FwJj14E UFL sample, while Ileret 2 corresponds to the pooled Ileret sample.

**Figure 4:**
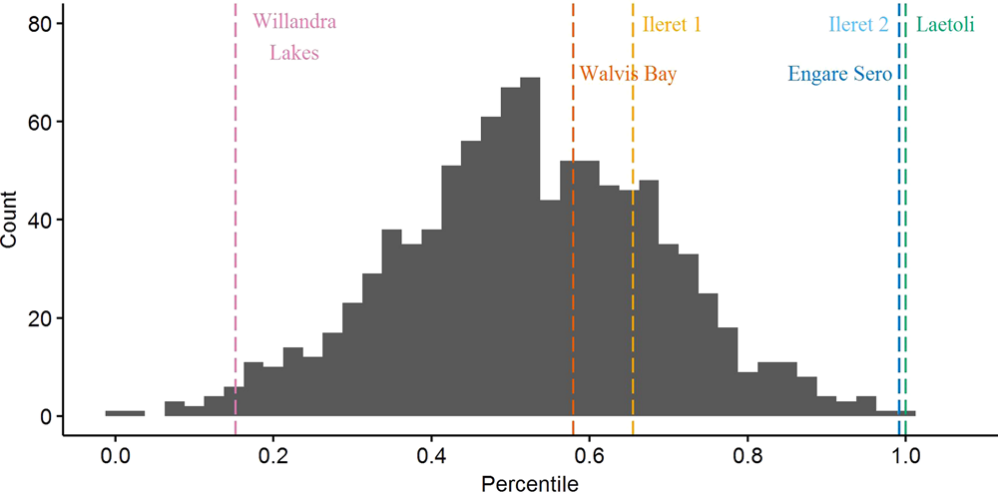
Bootstrapped human mean method quotients, using the Daasanach footprint length data. The Laetoli, pooled Ileret, and Engare Sero samples fall above the lower 95% of the distribution. The color and annotation scheme is as follows: orange (Ileret 1) = Ileret FwJj14E UFL; light blue (Ileret 2) = Ileret pooled; green = Laetoli; dark blue = Engare Sero; red = Walvis Bay; pink = Willandra Lakes. All the results are presented against a generalized distribution for convenience, but each sample was bootstrapped using the appropriate sample size in the actual statistical tests, as was also done for Figs 4–8 (see methods).

**Figure 5:**
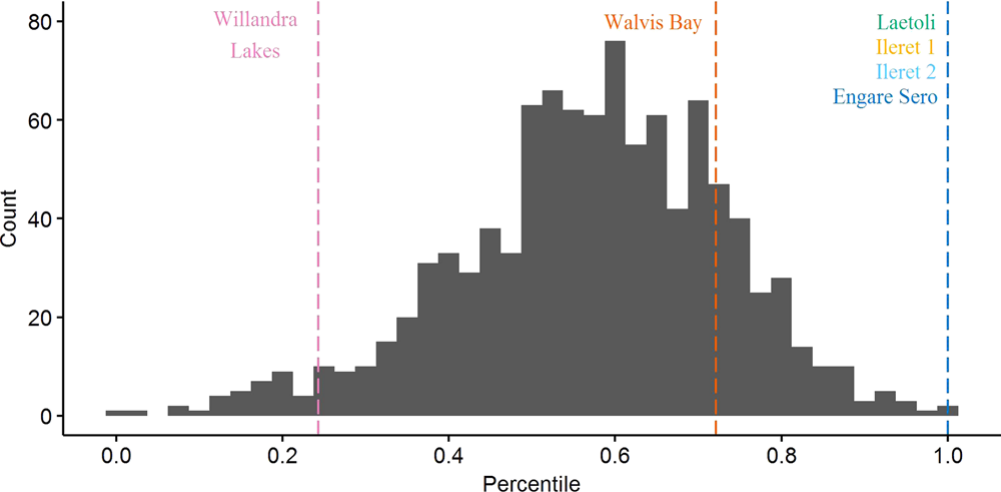
Bootstrapped human coefficients of variation (CVs), using the Daasanach footprint length data. The Laetoli, FwJj14E UFL, pooled Ileret, and Engare Sero samples fall above the lower 95% of the distribution. The color and annotation scheme is as follows: orange (Ileret 1) = Ileret FwJj14E UFL; light blue (Ileret 2) = Ileret pooled; green = Laetoli; dark blue = Engare Sero; red = Walvis Bay; pink = Willandra Lakes.

**Figure 6:**
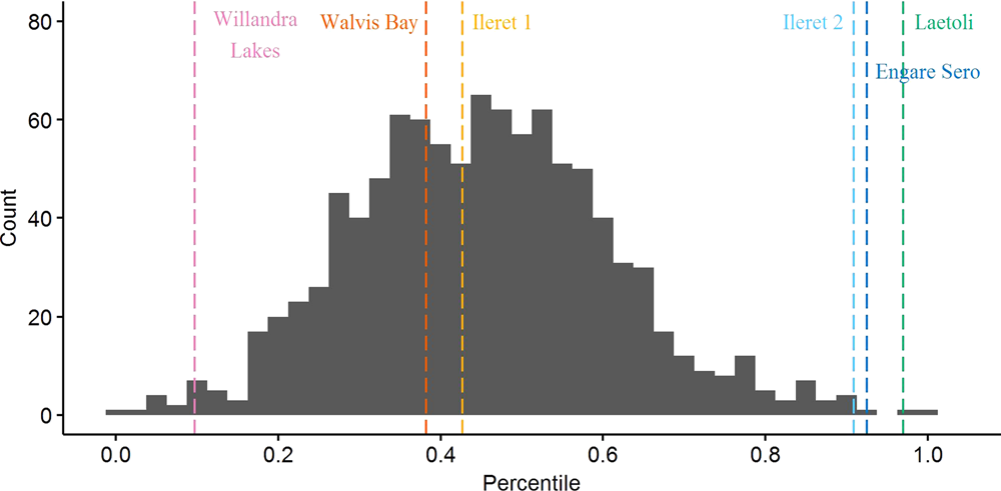
Bootstrapped human mean method quotients, using the US Army foot length data. The Laetoli sample is the only one that falls above the lower 95% of the distribution. The color and annotation scheme is as follows: orange (Ileret 1) = Ileret FwJj14E UFL; light blue (Ileret 2) = Ileret pooled; green = Laetoli; dark blue = Engare Sero; red = Walvis Bay; pink = Willandra Lakes.

**Figure 7:**
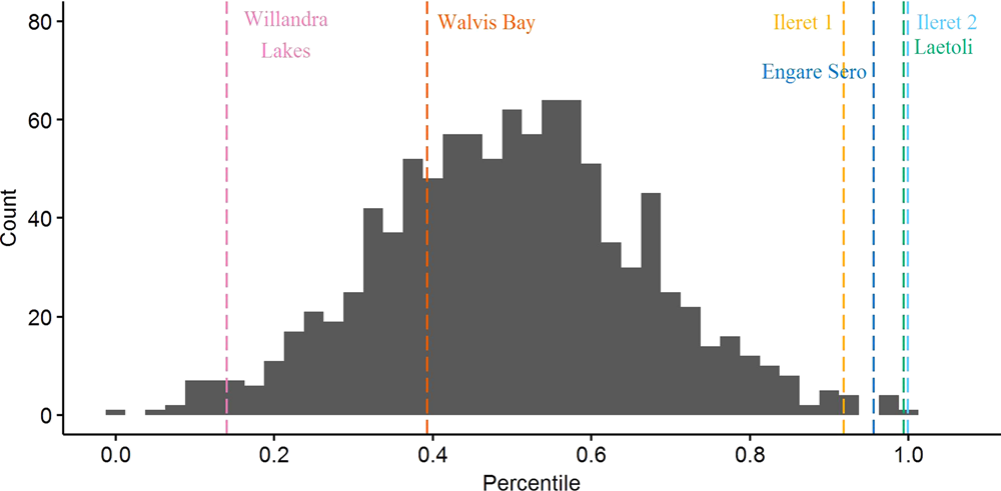
Bootstrapped human CVs, using the US Army foot length data. The Laetoli, pooled Ileret, and Engare Sero samples fall above the lower 95% of the distribution. The color and annotation scheme is as follows: orange (Ileret 1) = Ileret FwJj14E UFL; light blue (Ileret 2) = Ileret pooled; green = Laetoli; dark blue = Engare Sero; red = Walvis Bay; pink = Willandra Lakes.

**Figure 8:**
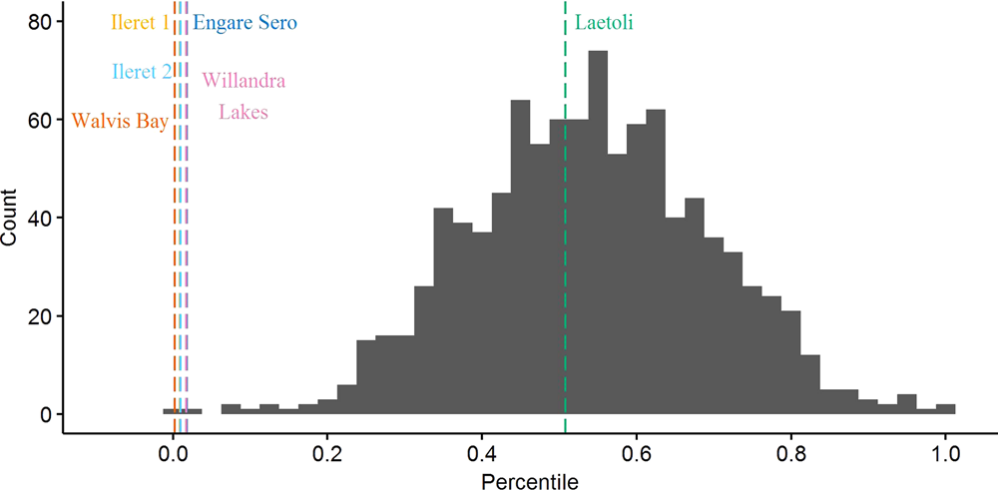
Bootstrapped mean method quotients from gorilla foot lengths. The Laetoli sample is the only one that falls within the upper 95% of the distribution. The color and annotation scheme is as follows: orange (Ileret 1) = Ileret FwJj14E UFL; light blue (Ileret 2) = Ileret pooled; green = Laetoli; dark blue = Engare Sero; red = Walvis Bay; pink = Willandra Lakes.

**Figure 9:**
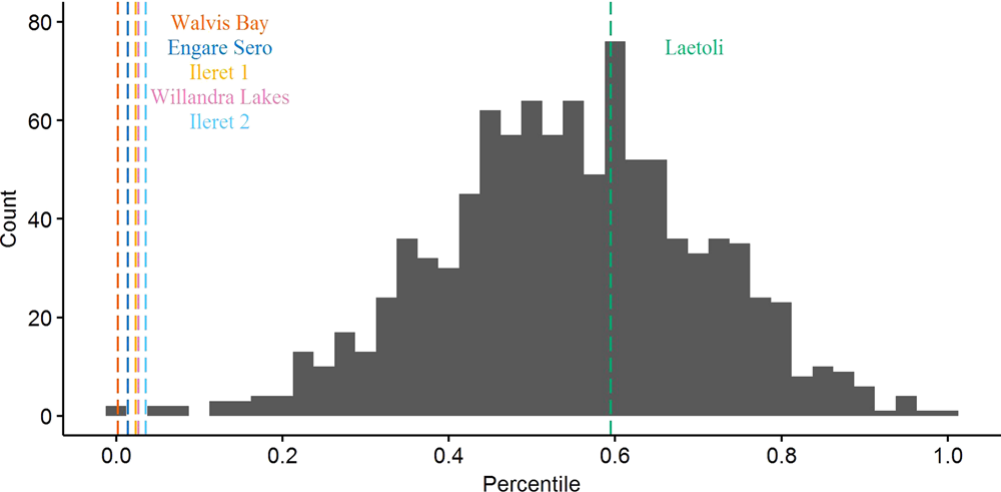
Bootstrapped CVs from gorilla foot lengths. The Laetoli sample is the only one that falls within the upper 95% of the distribution. The color and annotation scheme is as follows: orange (Ileret 1) = Ileret FwJj14E UFL; light blue (Ileret 2) = Ileret pooled; green = Laetoli; dark blue = Engare Sero; red = Walvis Bay; pink = Willandra Lakes.

On gross comparison of dimorphism patterns using the foot length data, the gorilla sample was noticeably more variable than any of the other comparative samples (Figs 2 and 3). The results of our bootstrapping analyses of dimorphism metrics can be seen in Table 3 and Figs 4–9. The simplest results are for the Laetoli footprints: this sample falls above 95% of the mean method quotients and CVs derived from each of the modern human samples (Table 3, Figs 3–6). Results for the Ileret assemblage are more complex. The pooled Ileret sample is also statistically more dimorphic than the Daasanach comparative sample (Table 3, Figs 5 and 6). However, the pooled Ileret mean method quotient (but not CV) falls within the lower 95% of the bootstrapped distributions from the much larger and more variable US Army sample (Table 3, Figs 3 and 4). The sample is therefore perhaps more variable than a mixed-sex modern human sample, but its pattern of dimorphism cannot be statistically distinguished from the pattern observed in a broad modern human sample. The Engare Sero sample also follows the same pattern, being distinct from the Daasanach sample in both the mean method and CV analyses, yet distinct from the US Army sample only in its CV (Figs 3–6). The Ileret FwJj14E UFL sample (presumably capturing members of the same population) has a CV that falls outside of the resampled Daasanach distribution, but a mean method quotient that falls comfortably within it (Table 3; Figs 5 and 6). Both the mean method quotient and the CV of the FwJj14E UFL sample fall within the lower 95% of the US Army sample (Table 3; Figs 3 and 4). The two remaining fossil track assemblages, from Willandra Lakes and Walvis Bay, fall within the lower 95% of both modern human distributions for both the mean method quotient and CV. These samples are therefore statistically indistinguishable from both modern human samples.

**Table 3:** Results of bootstrap analyses. On the left, the CV, Max/Min, R%, and mean method scores for the fossil footprints and comparative samples are presented.

Sample	*n*	Mean method quotient	CV	Max/Min	R%	US Army percentiles		Daasanach percentiles		*Gorilla* percentiles	
						Mean method quotient	CV	Mean method quotient	CV	Mean method quotient	CV
Ileret (FwJj14E UFL)	16	1.115	8.1	1.488	0.391	0.427	0.918	0.655	1.000	0.009	0.024
Ileret (pooled)	25	1.142	9.0	1.488	0.392	0.909	0.999	0.991	>1.000	0.010	0.036
Laetoli	6	1.194	11.6	1.364	0.314	0.970	0.994	>1.000	>1.000	0.508	0.595
Engare Sero	21	1.146	8.2	1.296	0.270	0.925	0.956	0.992	1.000	0.018	0.014
Walvis Bay	21	1.009	0.5	1.009	0.009	0.008	0.008	0.013	0.013	0.011	0.011
Willandra Lakes	7	1.069	4.6	1.111	0.106	0.097	0.140	0.152	0.243	0.017	0.027
*P*. *troglodytes*	37	1.108	6.3	1.090	0.249	0.232	0.257	0.480	0.719	<0.001	<0.001
*P*. *paniscus*	8	1.057	3.7	1.121	0.111	0.022	0.024	0.036	0.053	0.003	0.004
*Gorilla*	31	1.206	11.1	1.298	0.412	>1.000	>1.000	>1.000	>1.000	—	—
Daasanach	29	1.124	6.9	1.219	0.249	0.596	0.546	—	—	<0.001	<0.001
US Army	2000	1.121	6.8	1.507	0.407	—	—	>1.000	>1.000	<0.001	<0.001

On the right are the results of bootstrapping from the US Army, Daasanach, and *Gorilla* comparative samples. For each fossil footprint locality, a ‘fossil sample’ of equal size was drawn from each comparative sample 1000 times. We present the relative percentile of each fossil mean method quotient and CV within the bootstrapped comparative sample, which corresponds to the probability of drawing that quotient or CV from within the given bootstrapped sample. Hypothesis tests were one-tailed, so when a fossil sample falls at a percentile greater than 0.95 within the modern human distributions, that sample has a significantly higher level of dimorphism. Likewise, when fossil samples fall below 0.05 in the *Gorilla* distribution, this indicates a significantly lower level of size dimorphism.

Note: the sample sizes (column n) are the numbers used in the analysis, after the smallest, presumably juvenile, individuals had been removed (see text for explanation of methods).

Interestingly, when using the gorilla data as the comparative sample, all of the fossil footprint samples except for Laetoli fall below the upper 95% of bootstrapped mean method quotients and CVs (Table 3, Figs 7 and 8). The Ileret and Engare Sero samples, despite being statistically more variable and/or dimorphic than certain bootstrapped human distributions, are still significantly less variable/dimorphic than the gorilla sample. The same is true for the Willandra Lakes and Walvis Bay samples, leaving Laetoli as the only one that is statistically indistinguishable from the bootstrapped gorilla distribution.

## Discussion

We found the Ileret assemblages to be inconsistent with interpretations of an *A*. *afarensis*-like degree of sexual dimorphism for *H*. *erectus*. Dimorphism levels measured (using all measures of dimorphism) from the Ileret tracks fell well below the upper 95% of the resampled gorilla distribution (Fig. 5). However, when compared to modern human samples, the results are more equivocal. When we look at FwJj14E UFL, the footprints are statistically indistinguishable from modern humans, with the exception of a significantly greater CV compared with the Daasanach sample (Table 3, Figs 3–6). However, when we expanded the sample to include all known Ileret tracks, from multiple footprint surfaces, the CV and mean method quotients were significantly greater than those of the bootstrapped Daasanach sample (Figs 5 and 6). The CV also fell above the lower 95% of the bootstrapped US Army distribution, but the mean method quotient fell within the lower 95% (Figs 3 and 4). Further, both the mean method quotient and CV fell outside of the upper 95% of the gorilla sample. While it is true that statistical measures of dimorphism based on unknown sample distributions can, in some circumstances, overestimate dimorphism in samples with low levels of dimorphism^66^, our preferred interpretation of these results is that the pooled Ileret sample is intermediately dimorphic, more so than would be expected from an average mixed-sex modern human sample, but not as dimorphic as a mixed-sex gorilla sample. The relatively lower dimorphism estimates of the FwJj14E UFL sample could indicate that within-population size dimorphism was more moderate than the apparent species-level measures, or they could suggest that the FwJj14E UFL represents a sex-biased sample (see ‘Prospects for Further Research’). Regardless of which results we examine though, the Ileret and Laetoli footprint samples stand in contrast to each other. The Laetoli sample fell above the lower 95% for both human distributions (Figs 3–6), and comfortably within the upper 95% of the gorilla distributions (Figs 7 and 8). Therefore, our results fail to support the hypothesis of retention of high sexual dimorphism in *H*. *erectus*, but our results do support the hypothesis of a high, gorilla-like level of sexual dimorphism for *A*. *afarensis*. We note, incidentally, that the lower levels of dimorphism for the Ileret trackways provide circumstantial, but strong, support for the argument that these footprints represent *H*. *erectus* and not the contemporaneous *Paranthropus boisei*. Fossils of *P*. *boisei* are known to overlap in time with the Ileret tracks, but this species is highly dimorphic in cranial measures^24,72,73^, and therefore likely to have been similarly dimorphic in foot size. Other members of East African early *Homo*, *H*. *habilis* and *H*. *rudolfensis*, were presumably less dimorphic than *P*. *boisei* but their candidacies as potential Ileret trackmakers are undermined by conservatively estimated last appearance dates, which fall at 1.65 Ma and 1.78 Ma, respectively^74^, (although see citation^32^ for a potential later date for an early *Homo* specimen).

This result is generally consistent with the hypothesis of relatively human-like (but perhaps slightly higher) sexual dimorphism for *H*. *erectus*, rather than the greater levels of dimorphism that have been hypothesized by recent fossil discoveries and analyses^32^. In their announcement of the discovery of the KNM-ER 42700 cranium, Spoor and colleagues^32^ note that this skull is more similar in size to *H*. *habilis* than *H*. *erectus*, yet it appears to express many traits traditionally associated with the latter species, including sagittal keeling, cranial profile, and features of the temporal bone. They pointed to its similarity to the subadult Dmanisi cranium D2700; ironically, the later discovery of the Dmanisi cranium D4500 further added to the range of size variation in specimens attributed to *H*. *erectus*^75^. This subset of crania - KNM-ER 42700 and the Dmanisi sample – are largely responsible for the apparently elevated levels of body size dimorphism in *H*. *erectus*. However, it is important to note that the taxonomy of these specimens is not entirely clear: the taxonomic status of the Dmanisi sample is still in flux^75^, and many of the original authors who first analyzed KNM-ER 42700 recently proposed that it is actually not an adult *H*. *erectus* cranium^74^.

In a reassessment of general hominin body size based on postcranial data, Grabowski *et al*.^33^ also argued for a higher level of dimorphism in early *H*. *erectus* than observed in modern humans, which is not entirely discordant with the results found here. However, their estimates of dimorphism among East African and Georgian *H*. *erectus* are rather high, and would imply a level more similar to that observed in *A*. *afarensis*. These higher dimorphism estimates within the East African and Georgian *H*. *erectus* sample should be taken with a grain of salt, however, as Grabowski *et al*.^33^ note that they may be subject to error induced by the geographically and temporally disparate sample, as well as specimens (e.g., OH 28, BSN49/P27) that, although attributed to *H*. *erectus*, lack any cranial remains^76–78^ and could be attributed to different taxa^79^. When including specimens outside Africa and Georgia, Grabowski *et al*.^33^ estimated a level of sexual dimorphism more similar to that seen in modern humans. Those authors conclude that *H*. *erectus*, on a species-level scale, was likely more dimorphic than modern humans, and this result does not conflict with our findings here.

An advantage of using fossil footprints to evaluate size dimorphism is that one can avoid the problems potentially raised by lumping skeletal fossils that span broadly across time and space. This is especially true when examining *H*. *erectus*, a species whose fossil record spans more than 1.5 million years and multiple continents^3^. Even if time and space were constrained, and if one were to examine skeletal fossils from only the early Pleistocene of East Africa, then taxonomic variation becomes a confounding issue. There are currently at least three recognized species of early *Homo* known from this time (*H*. *habilis*, *H*. *rudolfensis*, *H*. *erectus*, and Neubauer *et al*.^80^ raise the possibility of a fourth), and significant debate over taxonomic variation in *H*. *erectus*^23,37,38,75,81–93^. An assemblage of skeletal fossils from this location and time period may sample multiple closely-related species with sufficiently similar postcranial anatomies that taxonomic assessment is impossible. For the Ileret footprints, a confident taxonomic assessment may be similarly difficult but, given the confined windows of space and time being sampled by these assemblages of footprints on the same land surfaces (about a day or less^55^), these data are probably much more likely to sample a single population of individuals from the same taxon. This may allow researchers an opportunity to examine population variation without the confounding effects of temporal and geographic variation. This is, in some ways, analogous to studying a single primate population in the wild, where parameters such as body size variation can be examined within a single breeding population. So, while we in no way discount the importance of skeletal fossils in understanding anatomical variation, we simply note that, when available, data from fossil footprints may offer relatively higher resolution snapshots of morphological variation within single populations.

Our results suggesting an intermediate level of dimorphism in *H*. *erectus* (less dimorphic than gorillas and *A*. *afarensis* but still slightly more dimorphic than modern humans) raise the possibility of anagenic change in *H*. *erectus*. If the Ileret footprints accurately represent *H*. *erectus* at 1.5 Ma, we may be capturing a glimpse of a transitional stage when the species lost much of the ancestral degree of sexual dimorphism, and is transitioning towards the relatively more monomorphic pattern seen in later populations of *H*. *erectus* (such as Zhoukoudian) and modern humans^94^. Notably, the Ileret footprint data support traditional interpretations of *H*. *erectus* as the first hominin to appear in the fossil record with a degree of sexual dimorphism that overlaps with modern humans, even if it is towards the upper extent of the modern human distribution.

Extending further, these results could have bearing on the evolution of social behavioral patterns in early hominins. However, Plavcan^12^ has outlined the limitations on resolution for coarse indicators, such as body size dimorphism, on complex socio-behavioral traits. He notes that, while body-size dimorphism always indicates strong male-male competition and polygyny, “the lack of dimorphism need not indicate a lack of male-male competition: low levels of dimorphism are not uniquely associated with any breeding system” (Plavcan^12^: 234). For *H*. *erectus* and the Ileret footprints, this represents a conundrum: how do we interpret a reduced level of dimorphism relative to an ancestral condition of high dimorphism? Under what circumstances, other than a changed social system, would strong dimorphism be selected away? Given what is currently known from the human fossil record, we struggle to conceive of an alternative to a change in the social system away from polygyny; if *H*. *erectus* had retained strong male-male competition and polygyny, we expect that, in absence of a strong selective force acting on dimorphism alone, that phylogenetic inertia would lead to the retention of body size dimorphism as well.

### Prospects for further research

Our pooled Ileret analysis, in which we expanded upon the Ileret FwJj14E UFL sample and included tracks of nine additional ‘individuals’ from other footprint surfaces, suggested that dimorphism estimates from the FwJj14E UFL sample may be slightly sex-biased. Analyses of the expanded sample generated dimorphism estimates that were slightly higher than those calculated from the FwJj14E UFL sample alone (although still low enough to be statistically similar to one of the modern human comparative samples, and statistically distinct from the gorilla sample). Based on body mass estimates that were derived from the Ileret footprints by Hatala *et al*.^56^, it seems more likely that the sample would be male-biased rather than female-biased. If the Ileret FwJj14E UFL sample is male-biased, then this raises intriguing questions about why such bias exists. Perhaps the footprints capture sex-biased foraging strategies in which groups of predominantly adult males foraged together in lake margin environments, as has been hypothesized previously^55–57,95^. For now this is still a somewhat speculative hypothesis, but future work aimed at developing methods for discerning specific behaviors from footprints could offer novel approaches for direct tests of social behavioral hypotheses using fossil ichnological data.

## Conclusion

The Ileret footprint assemblages (as well as other footprint sites) present researchers with remarkable windows into past events without the traditional confounding paleontological factors of time- and space-averaging. The large number of footprints within this sample provides an opportunity to test hypotheses about within-population sexual dimorphism, an anatomical trait that has the potential to be extremely informative with regards for hominin social structure. Our results find that the pattern of size dimorphism in the Ileret footprints is more consistent with the pattern seen in modern humans than with the much higher level of dimorphism seen in gorillas. The relatively high level of dimorphism seen in the Laetoli footprints (consistent with a gorilla-like level of dimorphism) provides a critical contrast for the Ileret sample. And while we are cognizant of the limitations of behavioral inference from an independent indicator such as body-size dimorphism, we parsimoniously interpret our results as suggesting that by 1.5 million years ago, at least one hominin species, most likely *H*. *erectus*, had transitioned away from polygyny.

### Significance

Understanding social behavior is critical for any animal yet interpreting behavioral patterns can be particularly difficult for extinct species. Social behaviors of fossil taxa are often inferred from their apparent levels of sexual dimorphism, as this characteristic often relates to social structure. However, fossil records that are typically fragmentary, and averaged across substantial amounts of both time and space, can make it difficult to accurately reconstruct this critical measure. We present the first use of fossil hominin footprints for assessment of sexual dimorphism, using the 1.5 Ma presumed *Homo erectus* footprint site near Ileret, Kenya. Our results indicate that these members of *Homo erectus* had a pattern of sexual dimorphism that was intermediate between earlier *Australopithecus* and later *Homo sapiens*. This study provides a novel approach for assessing sexual dimorphism in the human fossil record and gives the first view of within-population sexual dimorphism in African *Homo erectus*.

## Supplementary information

Appendix
