# Supplementary material for: Sexual dimorphism in Homo erectus inferred from 1.5 Ma footprints near Ileret, Kenya
Source: Sci Rep. 2019 May 22;9:7687. doi: 10.1038/s41598-019-44060-2 (PMC6531427; doi:10.1038/s41598-019-44060-2)
Supplement: Supplementary file 1 — Appendix [file 41598_2019_44060_MOESM1_ESM.docx]

**Supplement to:**

**Sexual dimorphism in *Homo erectus* inferred from 1.5 Ma footprints near Ileret, Kenya**

Brian Villmoare^1^, Kevin G. Hatala^2^, William Jungers^3,4^

1. Department of Anthropology, University of Nevada Las Vegas, Las Vegas, NV 89154-5003, brian.villmoare@unlv.edu

2. Department of Biology, Chatham University, Pittsburgh, PA, 15232,

kevin.g.hatala@gmail.com

3. Department of Anatomical Sciences, Stony Brook University, Stony Brook, New York, USA 11794-8081

4. Association Vahatra, BP 3972, Antananarivo 101, Madagascar,

williamjungers@gmail.com

**Supplementary text.**

To further explore the implications of our exclusion of hypothesized children’s footprints from calculations of dimorphism metrics in fossil footprint samples, we re-ran bootstrapping procedures in three different formats. First, we excluded the Laetoli G1 trackway from the Laetoli sample, in case that trackway actually represents a juvenile. Note that we did not exclude the G3 trackway, despite the fact that its average length (20.88 cm) is below the 22 cm threshold that we applied to the fossil *Homo sapiens* trackways. Exclusion in this case seemed inappropriate, as the application of the foot length/stature ratio observed in A.L. 288-1 (Jungers, 1988) results in a stature estimate of 129 cm, even more squarely in the range of skeletally-derived estimates for adult *A. afarensis* (McHenry, 1991). When excluding the G1 trackway, dimorphism estimates from the Laetoli sample were still significantly higher than those observed in the resampled Daasanach data set, and statistically indistinguishable from the resampled Gorilla data set (Table S1). However, although still on the high end, the dimorphism measures of this reduced Laetoli sample were not significantly different from those of the resampled US Army data set (Table S1). Therefore, dimorphism within a sample that includes only the Laetoli G2, G3, S1, and S2 trackways is still significantly greater, or on the higher end, of observed dimorphism in modern humans and statistically indistinguishable from that observed in gorillas.

In a second modification, we re-ran our analysis after removing the two Ileret trackways that had average footprint lengths below 22 cm. If we were to assume that the distribution of foot/footprint lengths (in a sense, the body size distribution) of *H. erectus* matched that observed in modern humans, then it is possible that these two trackways represent juveniles. When these trackways were excluded from our analysis, dimorphism measures in both of the Ileret samples (pooled and FwJj14E Upper Layer only) were still significantly lower from those in resampled gorilla data sets (Table S2). However, in this analysis, the dimorphism measures of both Ileret samples were mostly statistically indistinguishable from both modern human data sets. The only exception is that the CV of the pooled Ileret sample was still significantly higher than that of the resampled Daasanach distribution (Table S2). If the assumptions of this analysis were correct (that trackways with average track lengths of 20.5 and 21 cm represent juveniles and not adults), then this would indicate that dimorphism levels within the Ileret footprint samples were significantly lower than those observed among gorillas, and essentially indistinguishable from the levels observed in modern humans. Thus, rather than an intermediate level of dimorphism for *H. erectus*, this result would imply a human-like level. It is clear then that the inclusion or exclusion of these two trackways have significant bearing on the broader implications of our analysis. At the moment, until we know significantly more about *H. erectus* adult size variation and growth patterns, we are unable to say with any confidence that these trackways definitely represent juveniles. However, we recognize that the interpretations of our analysis may be different if a method is developed for definitively distinguishing large juvenile and small adult *H. erectus* footprints.

Finally, a third analysis followed a similar procedure to the analysis presented in the main text, but with all trackways from all fossil samples included. In other words, presumed juvenile tracks from the Engare Sero, Walvis Bay, and Willandra Lakes samples were still included. In this analysis, two of these three *H. sapiens* samples (Engare Sero and Willandra Lakes) exhibited levels of dimorphism that were significantly greater than those observed in either resampled modern human data set (Table S3). While the dimorphism measures of the inclusive Engare Sero sample fell comfortably within the range of measures observed in the resampled gorilla data set, the Willandra Lakes sample was significantly more dimorphic than the gorillas (Table S3). This result is clearly illogical, and demonstrates how mixed-age samples (adults and juveniles) can skew dimorphism metrics. The inclusive analysis of the Walvis Bay sample produced similar results as the more restrictive analysis presented in the main text, with dimorphism levels statistically indistinguishable from modern humans and significantly lower than those of gorillas (Table S3).

**Table S1. Analysis of dimorphism measures in reduced Laetoli sample.**

|  |  |  |  | |  | | US Army percentiles | | | Daasanach percentiles | | | *Gorilla* percentiles | | |
| --- | --- | --- | --- | --- | --- | --- | --- | --- | --- | --- | --- | --- | --- | --- | --- |
| Sample | Mean method quotient | CV | Max/Min | R% | | Mean method quotient | | CV | Mean method quotient | | CV | Mean method quotient | | CV |  |
| Laetoli (no G1) | 1.178 | 0.094 | 1.250 | 0.225 | | 0.904 | | 0.891 | 0.959 | | 0.971 | 0.426 | | 0.386 |  |

**Table S2. Analysis of dimorphism measures in reduced Ileret samples.**

|  |  |  |  | |  | | US Army percentiles | | | Daasanach percentiles | | | *Gorilla* percentiles | | |
| --- | --- | --- | --- | --- | --- | --- | --- | --- | --- | --- | --- | --- | --- | --- | --- |
| Sample | Mean method quotient | CV | Max/Min | R% | | Mean method quotient | | CV | Mean method quotient | | CV | Mean method quotient | | CV |  |
| Ileret  FwJj14E UFL (reduced) | 1.095 | 0.064 | 1.298 | 0.270 | | 0.122 | | 0.361 | 0.175 | | 0.714 | 0.002 | | 0.006 |  |
| Ileret pooled (reduced) | 1.118 | 0.074 | 1.326 | 0.290 | | 0.490 | | 0.775 | 0.727 | | 0.978 | 0.003 | | 0.002 |  |

**Table S3. Analysis of dimorphism measures from inclusive Engare Sero, Walvis Bay, and Willandra Lakes samples.**

|  |  |  |  | |  | | US Army percentiles | | | Daasanach percentiles | | | *Gorilla* percentiles | | |
| --- | --- | --- | --- | --- | --- | --- | --- | --- | --- | --- | --- | --- | --- | --- | --- |
| Sample | Mean method quotient | CV | Max/Min | R% | | Mean method quotient | | CV | Mean method quotient | | CV | Mean method quotient | | CV |  |
| Engare Sero (inclusive) | 1.190 | 0.120 | 1.978 | 0.601 | | >1 | | >1 | >1 | | >1 | 0.780 | | 0.219 |  |
| Walvis Bay (inclusive) | 1.113 | 0.065 | 1.219 | 0.204 | | 0.382 | | 0.393 | 0.579 | | 0.721 | 0.002 | | 0.002 |  |
| Willandra Lakes (inclusive) | 1.548 | 0.191 | 1.875 | 0.544 | | >1 | | >1 | >1 | | >1 | >1 | | >1 |  |
